# Hospital Reservoirs of Multidrug Resistant *Acinetobacter* Species—The Elephant in the Room!

**DOI:** 10.3389/bjbs.2023.11098

**Published:** 2023-03-20

**Authors:** S. Fahy, J. A. O’Connor, B. Lucey, R. D. Sleator

**Affiliations:** ^1^ Department of Clinical Microbiology, Mercy University Hospital, Cork, Ireland; ^2^ Department of Biological Sciences, Munster Technological University, Cork, Ireland

**Keywords:** *Acinetobacter*, environmental screening, multi-drug resistance, indicator organism, microbiological decontamination

## Abstract

Environmental contamination is estimated to contribute to up to 20% of all hospital acquired infections. *Acinetobacter baumannii* is an example of one the most prevalent opportunistic pathogens causing severe and persistent infections in immunocompromised patients. It has proven ability to form biofilms, has significant associated multi-drug resistance and is able to transfer mobile genetic elements to other clinically relevant pathogens. All of these factors point to a definite utility of *A. baumannii* as an indicator organism for effectiveness of decontamination regimens as well as environmental screening. There is an increased cost, both financial and clinical, associated with multi drug resistant organisms, carbapenem resistant *A. baumannii*. With a dearth of new antimicrobials in development, now is the time to radically transform and lead the introduction of scientifically based environmental screening and microbiological verified decontamination to control the dissemination of further resistance.

## Introduction

There is increasing evidence to suggest that environmental contamination of hospital surfaces contributes significantly to the spread of pathogens, including Multi-Drug Resistant Organisms (MDROs) such as carbapenemase producing Gram-negative *Enterobacterales* (1). Indeed, there is a growing body of evidence to suggest that hospitals and long-term care facilities, are a rich reservoir of MDROs which have the potential to infect vulnerable patients (2). Screening and infection control measures for such organisms are inconsistent at best, with little or no official guidance as to what preventative screening should be implemented. The National Health Service’s (NHS) specifications for Cleanliness, for example, outlines only general monitoring of hospital environments to be assessed by visible audit, with no recommendation for microbiological screening (3). Similarly lacking, the Irish Health Service Executive’s (HSE) national cleaning standard is based on a 2005 evaluation (4). This current deficit in environmental screening is likely attributable to the extra financial cost of such screens (5) and over-stretched infection prevention and control (IPC) teams, together with the already significant burden of an increasing workload of greater complexity in understaffed microbiology laboratories (6). Nonetheless, environmentally acquired pathogens continue to pose a real and present risk in the form of environmental reservoirs that all too often go unnoticed (7). Immunocompromised patients and those with increased lengths of hospital stays are most at risk (8). The focus of the current review is to highlight the rising problem of one such bacterial strain, using multi drug resistant *Acinetobacter baumannii* as a model organism of environmental contamination. *A. baumannii* represents an ideal indicator organism of such environmental reservoirs as it is stress resistant (9), antibiotic resistant (10), a widely attributed opportunistic pathogen (11) and a definite source of virulence genes that have the potential to be acquired by other, clinically relevant pathogens (12).

## Impact of Hospital Acquired Infections

A point prevalence study on Hospital Acquired Infections (HAIs) conducted by the European Centre for Disease Prevention and Control (ECDC) across 33 countries in 2011/12, reported a 5.5% HAI prevalence for Europe, 5.9% for the United Kingdom and 5.2% for Ireland (13). This was updated further in 2017 with a Eurosurveillance study carried out on hospitals and long-term care facilities (LTCF). The HAI prevalence on this occasion was 6.1% for Ireland, 6.4% for England and 5.6% for the UK; indicating that both countries are above the previously reported European average. The prevalence of HAIs was highest in intensive care units (24%) and surgical wards (9.1%). The top four types of HAIs documented are pneumonia (28.9% of all HAIs), surgical site infections (18.1%), urinary tract infections (1.5%), and bloodstream infections (9.9%) (14). The economic cost of these HAIs was estimated to be ∼€7 billion per annum in Europe alone, a decade ago (13). The direct risk posed to a patient by a lack of environmental cleanliness, particularly in terms of microbial contamination, is difficult to determine. There are, however, strong links with cross-transmission of pathogens from healthcare workers’ hands, or from indirectly touching contaminated surfaces (15). Herein, we focus on *A. baumannii* as an indicator organism of environmental contaminants, which in turn represent a significant reservoir of HAIs.

## Survivability of *Acinetobacter baumannii*

*A.baumannii* are non-motile, non-fastidious, non-fermentative, catalase-positive, oxidative-negative Gram-negative coccobacilli that are most often associated with aquatic environments (16, 17). Responsible for approximately 2%–10% of all Gram negative hospital acquired infections (18), *A. baumannii* in particular is an extremely stress resistant opportunistic pathogen, with the ability to survive for prolonged periods in the hospital environment, and on medical devices (17). A key virulence and stress associated factor in *A. baumannii* are OmpA, a member of the outer membrane proteins (OMPs) which causes mitochondrial dysfunction, leading to cell apoptosis (9). The same OmpA surface protein is responsible for the formation of biofilms, a key survival mechanism of *Acinetobacter baumannii* in the environment (19). The ability of *Acinetobacter* to colonise and produce biofilms on surfaces leads to chronic and persistent infections, antibiotic resistance, and increased survival in hospital environments (20). Factors controlling biofilm formation include nutrient availability, the presence of pili and outer membrane proteins and macromolecular secretions (19). It has been observed that plasmids and biofilm structure/functions are intertwined through complex interactions, with recent research revealing that lateral gene transfer and biofilm formation are connected processes (21). The horizontal transfer of mobile genetic elements (MGEs) is enhanced by biofilms promoting plasmid stabilisation with horizontal transfer rates being higher in biofilm communities compared with those in planktonic states (21). Biofilm related, ventilator-associated *Acinetobacter* pneumonia can also be extremely resistant to antibiotics, representing a serious challenge to the clinical management of infections (22). A study by Perez et al. at a New Jersey hospital showed an increase in carbapenem resistant *A. baumannii* (CRAB) during a surge in COVID-19 which resulted in an increase in ventilator dependent patients (23). Another US study demonstrated that patients colonised with *A. baumannii* on admission to ICU were 15.2 times more likely to develop a subsequent positive clinical culture and are 1.4 times more likely to die during hospitalisation (24), highlighting the pressing need to definitively ascertain the link between environmental reservoirs and patient transmission potential.

## *Acinetobacter baumannii*—Mechanisms of Resistance

In addition to the direct threat posed by *A. baumannii* as an opportunistic pathogen, it also represents an important reservoir of stress resistance genes and virulence associated factors, such as *ompA*, which when acquired by lateral gene transfer can significantly bolster survival and virulence potential when expressed phenotypically by other clinically relevant pathogens (12). In addition to environmental stress tolerance, *A. baumannii* has also been shown to be extremely antibiotic resistant, with resistance to third generation cephalosporins and carbapenems being reported as early as the 1980s; a phenotype which is known to result from a variety of different genetic mechanisms (10). Horizontal gene transfer can occur by three mechanisms known as transformation, transduction, and conjugation (25). Mobile genetic elements (MGEs) such as bacteriophages, plasmids and conjugative transposons are used during horizontal gene transfer of foreign DNA between bacteria in the same environment (26), with conjugation being the driving force in acquisition of exogenous DNA in *Acinetobacter* spp. (11)*.* blaOXA_23_, an acquired subgroup of carbapenem-hydrolysing class D β-lactamases (CHDLs), can be inserted both in the chromosome and in plasmids and can inactivate carbapenems, is now widely distributed in *A. baumannii* (27). A 2014 report by La et al. describes the unusual detection of a blaOXA_23_ in a clinical *Escherichia coli* isolate (28). This was the first description of a plasmid borne blaOXA_23_ in *E. coli* and was detected as part of a national surveillance of non-carbapenem susceptible Enterobacterales*.* Comprehensive PCR sequencing confirmed the blaOXA_23_ had 100% identity to the *A. baumannii* blaOXA_23._ Southern blot hybridization located the blaOXA_23_ on a 50-kb plasmid, which was shown to be conjugative (28). This concerning discovery re-emphasises the importance of controlling carbapenemase dissemination from non-carbapenemase producing bacteria. Huang et al. reported isolating *E. coli*, *Citrobacter freundii* and *A. baumannii* from the same patient. All isolates had acquired a *bla*_NDM-1_ gene, a known carbapenemase producing gene, suggesting gene transfer in same environments (29). A Swiss study also reports the presence of the *bla*_NDM-1_ gene in Enterobacterales located on conjugative IncA/C- or IncF-type plasmids. Part of ISAba125, an insertion sequence which causes resistance to third generation cephalosporins and located upstream of ampC gene was present on the plasmid. ISAba125 has previously been identified in *bla*_NDM-1_ negative *A. baumannii*, suggesting that the original source was *A. baumannii* (30). There is a possibility that horizontal transfer of chromosomally located resistance genes occurs more commonly than previously thought. Furthermore, Maeusli et al showed environmental *Acinetobacter baylyi* is capable of transferring plasmids harbouring resistance genes to clinical isolates of *E. coli* on lettuce leaves (31). Interestingly, the same strain of *E. coli* could colonise the gut microbiome of mice with *in vivo* transfer of the plasmid to *Klebsiella pneumoniae* within 5 days (31).

It has been shown that most chronic infections are now biofilm related (32) with bacteria residing in mature biofilms being better protected against antibiotic exposure than their free-living counterparts. Plasmids enhancing survivability of their hosts will therefore enhance their own persistence (21). Dense communities of biofilms expediting the spread of MGEs through a spatial and structural advantage is thought to increase conjugation in biofilm communities (21). Plasmids which are maintained through high transfer frequencies, may only be able to persist in biofilms (33) as conjugation occurs at higher frequencies in biofilm communities. As *A. baumannii* is an established biofilm forming bacterium, it was proposed that the transfer of *bla*_NDM-1_ plasmids could potentially occur more readily in this environment (34). Three clinical and environmental *Enterobacterales* strains carrying *bla*_NDM-1_ were mated to form *E. coli* J53 *bla*_NDM-1_ transconjugants. Donor biofilm *A. baumannii* showed the successful uptake of the *E. coli* J53 *bla*_NDM-1_ transconjugants in two of the three strains, demonstrating the potential for NDM spread in clinical and environmental settings (34).

Antibiotic release into the environment *via* human, veterinary and agricultural waste likely represents a significant contributor to the emergence and maintenance of resistance (35). Antibiotic concentrations vary in natural environments, including wastewater from pharmaceutical industries or hospitals, with fluroquinolones frequently reaching the highest levels in milligrams per millilitre concentrations (35). Even at the very low antibiotic concentrations which are present in natural environments, it is sufficient for the maintenance of pre-existing resistant bacterial strains, along with the *de novo* selection of new mutants (35). A wastewater treatment study by Higgins et al. in Zagreb, Croatia, over a 1-year period concluded that 102/119 *A. baumannii* isolates retrieved from various stages of wastewater treatment were carbapenem resistant (36). Viable *A. baumannii* complex isolates are constantly being emitted *via* effluent into the environment and can survive the technological process of anaerobic mesophilic sludge digestion (36). This further supports the potential community dissemination of antibiotic resistance. Antibiotic resistant *Acinetobacter* spp. including *A. baumannii* have also been recovered from vegetables which may provide an additional route into hospitals (37).

## Environmental Screening in a Hospital Setting

**FIGURE 1:**
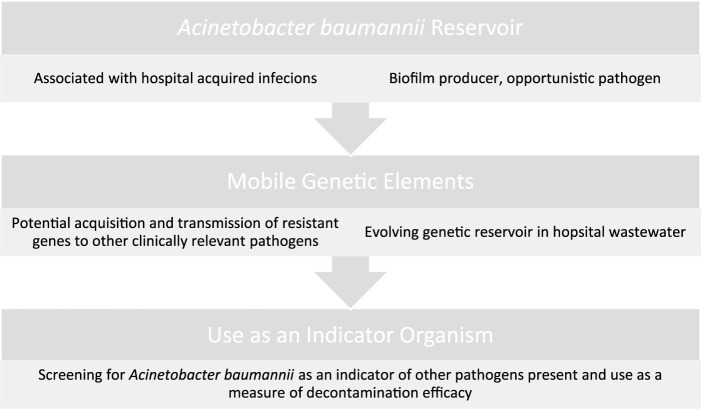
Threats and usefulness of *Acinetobacter baumannii* as an environmental contaminant.

It is estimated that up to 20% of HCAIs can be attributed to environmental contamination (38). Generally, hospital environments are only sampled in response to an outbreak with routine sampling not usually indicated for healthcare environments. Hospital surface environments act as a reservoir for pathogens where prior room occupants shed organisms onto their environment, posing a risk to the next occupant (see Figure 1). Where the cleaning is sub-optimal, patients have an increased risk of acquiring a HAI when a previous patient has been colonised (39). Most hospitals are currently only visually assessed for cleanliness but include no microbiological auditing. The Health Service Executive (HSE) currently advises microbial settlement monitoring by passive sampling and the efficacy of cleaning should be monitored microbiologically using contact media containing neutralisers (4). Public Health England guidelines, published in 2020 on environmental sampling outlines that risk assessments must be carried out in healthcare facilities, cleaning tasks are documented and the effectiveness of cleanliness is to be monitored [British Standards Institution (40)]. The document states that routine sampling is not usually indicated. There are however guidelines available should screening be required (3). A review of European guidelines in controlling multi-drug resistant Gram-negative bacteria contain broad areas of agreement, however, there is discordance between guidelines. All guidelines agree environmental screening is to be carried out in outbreak settings only, while there are disagreements on the approach to healthcare worker screening during an outbreak (41). Rawlinson et al. reviewed current environmental monitoring of hospital surfaces in non-outbreak settings and how best to sample areas (42). Their results showed that swabs are better than contact plates when recovering Gram-negative pathogens, while still reporting on the optimal selection of culture medium to use when conducting *Acinetobacter* screening. There are a range of factors that can introduce sampling bias when screening an environment; including the level of contamination, whether the sample is taken from a wet or dry surface, pressure/contact time, and post-test processing. The microbial recovery from surfaces was improved by pre-moistening for all swab type (43), with increases in recovery from 57.5% on dry surfaces to 83.4% with pre-moistening of the same surface (44). When processing environmental samples, the choice is dependent on the target organism, cost, and time available. The choice of extraction solution was found to play a major role in recovering environmental pathogens when using culture methods (45). Furthermore, it has been shown that vortexing can increase recovery yield when using flocked swabs (46). Colony forming units (cfu) used when reporting environmental swabs often do not reflect the true risk to the patient, as it has been shown that surfaces with the highest bioburden are not always the surfaces with the most multi-drug resistant organisms (MDROs), which are of greater clinical concern (47). This can be seen when environmental screening is traditionally carried out for Gram-positive isolates on frequently touched surfaces. There is a lack of available information on how to individually target specific organisms when disinfecting hospital environments, a 2015 study by Havill et al. demonstrates the persistence of *A. baumannii* complex: after four rounds of manual cleaning and disinfection with a bleach solution, 25% of rooms were still contaminated with *A. baumannii* (48). Speculation on environmental contamination needs to become scientifically standardised (5).

A 2015 study by Simor et al. described a multidrug resistant *A. baumannii* (MDRAB) outbreak in a hospital burn unit, whereby 13% of patients acquired a MDRAB over a 15-month period. Environmental screening recovered MDRAB from healthcare workers hands and the hospital environment. Strict infection control and meticulous environmental cleaning contained the outbreak (49). A study by Al-Hamad et al., using pulse-field gel electrophoresis (PFGE) identified a pair of clinical and environmental *bla*_NDM-1_ carbapenem resistant *Acinetobacter baumannii* (CRAB) isolates from a surgical ward in Saudi Arabia (50). This is particularly concerning and highlights the importance of a targeted pathogen specific screening and decontamination protocol.

## Carbapenem Resistance in *Acinetobacter* spp.

It has been shown that CRAB ICU infections are associated with increased rates of mortality in contrast with other pathogens being associated with increased length of stays and hospital costs (51). It is concerning that only six new antibiotics were released for use between 1985 and 2015 and resistance has already been observed to each of these (52) thus, the importance of following the Association of Professionals in Infection Control and Epidemiology (2010) guide to the elimination of MDRAB transmission in healthcare settings has not been overstated (53). These steps include risk assessment, infection surveillance, strict adherence to hand hygiene, antibiotic stewardship, outbreak recognition and environmental decontamination. The increasing occurrence of MDRAB among vulnerable patients highlights the importance of surveillance as a critical component aimed at preventing the spread of antimicrobial resistance (54). Early detection of CRAB outbreaks is key in preventing uncontrolled outbreaks (55). A 2019 study by Yamamoto et al. discusses the potential for rapid molecular identification of CRAB isolates in vulnerable patients (55). In that study, loop-mediated isothermal identification (LAMP), which is a rapid molecular diagnostic assay, was employed to help control an ICU outbreak. The use of this intervention resulted in the prevalence decreasing from 35.2 per 1,000 patient days in the outbreak phase to 20.9 in the active intervention phase, owing to earlier detection of CRAB facilitating earlier infection control measures. In areas with extensive amounts of environmental contamination, strict infection control measures including isolation, active surveillance cultures and daily environmental cleaning minimises the spread as was seen in a burn unit with an active healthcare associated infection (HCAI) outbreak of MDRAB, showing a decrease in spread of 88% during the period of intervention (56). Another report where patient surroundings were heavily contaminated showed that the burden of contamination correlated with the patient colonisation load, further demonstrating the need to monitor the environment as a CRAB reservoir (39).

## Future Prospects

The increase in antibiotic resistance, together with uncertain prospects of new antibiotic development, means that a clear-cut environmental plan is urgently required. A single resistant infection has been estimated to cost approximately €8,500 to €34,000 more than a non-resistant infection, by the Organisation for Economic Cooperation and Development (OECD) (57). Clinical microbiology laboratories are an essential subspecialty in identifying environmental reservoirs and characterising antibiotic profiles of multi drug resistant pathogens. Clinical laboratories contribute directly to patient care at an individual and institutional level in the management of infection (6). A universally standardised policy for pathogen specific decontamination of hospital environments paired with an environmental molecular panel on the ESKAPE (*Enterococcus faecium*, *Staphylococcus aureus*, *Klebsiella pneumoniae*, *A. baumannii*, *Pseudomonas aeruginosa* and *Enterobacter* spp.) six pathogens used to screen high touch/high risk areas. Increased patient screening on admission is another beneficial method of identifying MDROs that may have potential for widespread transmission. The vital role cleaning staff play in infection prevention and control is not to be understated. Staff should be trained accordingly to understand the importance of microbiological decontamination as opposed to visible cleanliness.

## Recommendations

More needs to be done to screen for MDRO in the hospital environment, particularly to identify and effectively decontaminate environmental reservoirs. The only steadfast way of reliably doing this is through microbiological screening and assessment of cleaning protocols. We strongly suggest that this microbiological screening of the hospital environment should use A. *baumanii* as an indicator organism for the presence of the other ESKAPE organisms. This is primarily due to the triple threat of *A. baumannii* as an opportunistic pathogen, a biofilm former and as an environmental reservoir of mobile genetic elements. Biomedical scientists are demonstrated leaders when it comes to developing and guiding appropriate infection prevention and control measures and central to good antimicrobial stewardship, although accustomed to being in the back room. However, the urgency of preserving what value remains on antibiotics suggests that as a profession we need to take a lead when it comes to the control of *A. baumannii* and other nosocomial pathogens.
